# Deconstructing depression by machine learning: the POKAL-PSY study

**DOI:** 10.1007/s00406-023-01720-9

**Published:** 2023-12-13

**Authors:** Julia Eder, Lisa Pfeiffer, Sven P. Wichert, Benjamin Keeser, Maria S. Simon, David Popovic, Catherine Glocker, Andre R. Brunoni, Antonius Schneider, Jochen Gensichen, Andrea Schmitt, Richard Musil, Peter Falkai, for the POKAL Group, Tobias Dreischulte, Peter Henningsen, Markus Bühner, Katharina Biersack, Constantin Brand, Vita Brisnik, Christopher Ebert, Feyza Gökce, Carolin Haas, Lukas Kaupe, Jonas Raub, Philipp Reindl-Spanner, Hannah Schillock, Petra Schönweger, Victoria von Schrottenberg, Jochen Vukas, Puya Younesi, Caroline Jung-Sievers, Helmut Krcmar, Karoline Lukaschek, Kirsten Lochbühler, Gabriele Pitschel-Walz

**Affiliations:** 1 Department of Psychiatry and Psychotherapy, LMU University Hospital, LMU Munich, Nussbaumstrasse 7, 80336 Munich, Germany; 2 Graduate Program “POKAL - Predictors and Outcomes in Primary Care” (DFG-GrK 2621, Munich, Germany; 3 International Max Planck Research School for Translational Psychiatry (IMPRS-TP), Munich, Germany; 4 Max-Planck Institute of Psychiatry, Munich, Germany; 5 Department of Psychiatry, Faculty of Medicine, University of São Paulo (FMUSP), São Paulo, SP Brasil; 6 Institute of General Practice and Health Services Research, School of Medicine, Technical University Munich, Munich, Germany; 7 Institute of General Practice and Family Medicine, Ludwig-Maximilians-University Munich, Munich, Germany; 8 Laboratory of Neuroscience (LIM27), Institute of Psychiatry, University of São Paulo, São Paulo, Brazil; 9 Oberberg Specialist Clinic Bad Tölz, Bad Tölz, Germany

**Keywords:** MDD, Phenotyping, Machine learning, Biological psychiatry

## Abstract

Unipolar depression is a prevalent and disabling condition, often left untreated. In the outpatient setting, general practitioners fail to recognize depression in about 50% of cases mainly due to somatic comorbidities. Given the significant economic, social, and interpersonal impact of depression and its increasing prevalence, there is a need to improve its diagnosis and treatment in outpatient care. Various efforts have been made to isolate individual biological markers for depression to streamline diagnostic and therapeutic approaches. However, the intricate and dynamic interplay between neuroinflammation, metabolic abnormalities, and relevant neurobiological correlates of depression is not yet fully understood. To address this issue, we propose a naturalistic prospective study involving outpatients with unipolar depression, individuals without depression or comorbidities, and healthy controls. In addition to clinical assessments, cardiovascular parameters, metabolic factors, and inflammatory parameters are collected. For analysis we will use conventional statistics as well as machine learning algorithms. We aim to detect relevant participant subgroups by data-driven cluster algorithms and their impact on the subjects’ long-term prognosis. The POKAL-PSY study is a subproject of the research network POKAL (Predictors and Clinical Outcomes in Depressive Disorders; GRK 2621).

## Introduction

Unipolar depression is a common disorder worldwide, with a 12-month prevalence of 4.6% [1]. Unfortunately, only 41% of the affected patients who wanted treatment for their disorder (71%) receive treatment that meets minimum standards [1]. At the same time, depression is one of the most debilitating diseases and imposes a significant challenge to both individuals and society. According to WHO, depression causes 6% of the burden of disease in Europe, if measured in disability-adjusted life years (DALYs) [2, 3]. The worldwide point-prevalence of a depressive disorder was 9.8% (95% CI: 6.7–14.1%) within the years 1994–2003 and has risen to 15.4% (CI: 12.9–18.3%) between the years 2004 and 2014 [4, 5].

Moreover, a patient's psychiatric history was found to be correlated with higher age at graduation on the one hand and unemployment on the other [6]. Early onset of depression was associated with dropout from secondary education [7]. In the context of employment, 5.1% of all days absent from work are attributed to depressive disorders and thus, along with chronic pain disorders, have the greatest negative effect on job attendance [8]. The prognosis is worse the more time passes until diagnosis and adequate therapy is established [9]. Currently, only 55% of depressed patients seeking treatment in primary care settings are detected. Especially, if there are other somatic comorbidities present, mental health disorders are underdiagnosed [10, 11]. While also most psychiatric care is delivered in primary care settings [12], delayed diagnosis and inadequate treatment leads to an increased socioeconomic burden and outcome [13].

Primary care physicians could screen for unipolar depression with the Patient Health Questionnaire 9 (PHQ-9) [14], as it is also recommended by the U.S. preventive task force [15]. Since the PHQ-9 is a self-rating questionnaire, heterogeneous response behavior can’t be avoided, and more extensive diagnostics are needed to determine whether someone has depression [16]. Although diagnostic accuracy is typically higher for clinically experienced psychiatrists, a thorough clinical interview still requires time and experience. Currently, there are no established biological markers that could facilitate and accelerate the diagnostic process for primary care physicians.

A lack of fast, cheap and efficient diagnostic tools in primary care might be a cause for late treatment and detection of unipolar depression. “Unipolar depression” as a clinical phenomenon probably combines numerous biological phenotypes, which significantly complicates the detection of biomarkers using classical statistical methods [17].

### Potential somatic factors and biological predictors

#### Weight gain and metabolism

Considering that primary care physicians typically focus on somatic symptoms, it is important to note that unipolar depression is associated with a range of comorbidities such as increased cardiovascular risk, adiposity, dyslipidemia, as well as high blood pressure and hyperglycemia, resulting in an increased cardiovascular risk [18]. These somatic symptoms might be the main focus of primary care physicians when diagnosing and treating patients, making it even more crucial to consider the possibility of depression and its potential impact on overall health. In fact, the presence of a metabolic syndrome, which is defined as a combination of obesity, low HDL cholesterol, high blood pressure, and elevated blood sugar levels, has been linked to unipolar depression [19]. The metabolic dysregulation in unipolar depression is associated with immunologic changes, which might only affect a clinical subgroup of patients, namely patients with higher childhood trauma scores and rather atypical features of unipolar depression [20, 21]. The link between increased body fat percentage and chronic low-level inflammation [22], childhood trauma, depression, and low physical activity is well documented [22–24]. Increased body weight is associated with decreased well-being, vitality, and quality of life [25]. Weight gain may be followed by the development of metabolic syndrome [26], which increases the risk of cardiovascular disease, autonomous dysregulation [27] and type II diabetes. Patients with unipolar depression have a higher likelihood to suffer from hypercholesterolemia and triglyceridemia compared to their non-affected peers. This association was especially present in women and in patients older than 40 [28]. A lower expression of brain-derived-neurotropic-factor (BDNF), which is associated with mood disorders like unipolar depression, could be observed in mice that were put on a hypercaloric diet [29]. Another hormone that might play an important role in the context of depression and a dysregulated metabolism is Insulin-like Growth Factor—1 (IGF-1). The expression of IGF-1 is influenced by nutrition, BMI and insulin levels, as well as age, sex, and ethnicity. While IGF-1 is mainly known for being a growth hormone that also increases testosterone production [30], evidence further suggests that IGF-1 plays an essential role in dementia, cardiovascular, and metabolic diseases [31, 32]. Elevated IGF-1 levels may increase the risk of developing depression [33, 34], but seems to also mediate the rapid antidepressant effect of ketamine [35, 36].

#### Hypothalamic–pituitary–adrenal-axis

Alterations in the hypothalamic–pituitary–adrenal axis (HPA axis) are involved in neuropsychiatric disorders and have long been a focus of depression research [37]. The dysregulation of the HPA axis is associated with a lack of response to therapy as well as decreased cognitive performance [38]. To date, there is no specific drug targeting the components of the HPA axis that is currently approved and no test that is routinely used in the psychiatric setting to identify patients with HPA-dysregulation [39]. Studies showed that such patients could profit from a normalization of the cortisol rhythms [40].

#### Heart rate variability

In conjunction with the HPA axis, the autonomic nervous system (ANS) forms an extensive and complex network of integrated communication that regulates the body’s physiological homeostasis [41]. The heart rate variability (HRV) is seen as a reflection of the current stress state of the ANS [42, 43]. Chronically reduced HRV indicates autonomic imbalance [44], which is observed in patients suffering from depression or anxiety [45]. Numerous research findings suggest that reduced HRV predicts poor cardiovascular health outcomes, both in heart-healthy populations and in populations with pre-existing cardiovascular disease [46]. The HRV is a trans-diagnostic factor that can be associated with various stress-related conditions and behavioral factors, as well as somatic diseases [47]. While listening to music could increase HRV in stressed participants this effect could not be observed in participants with depression [48], which might be due to reduced resilience, meaning that the autonomous nervous system cannot respond with increased parasympathetic activity [49]. Higher HRV has been associated with psychological flexibility, emotional self-regulation, empathy, and social engagement [50, 51]. HRV could be found to be an important mediator between depression, autonomic dysregulation, and chronic cardiovascular disease [45, 52–54]. A meta-analysis concluded that parasympathetic activity is negatively correlated with inflammation [55], showing again a connection to metabolic disbalances [56]. The complicated dynamic interactions between inflammation and other relevant neurobiological correlates of depression is an indication that a parameter such as the HRV could be an important therapeutic marker for the evaluation of future therapeutic strategies, as well as a diagnostic tool in depressive disorders [57].

#### Inflammation and the dysregulation of the immune system

Excessive or persistent activity of proinflammatory cytokines disrupts numerous neuronal functions, including impairment of neurotransmitter signaling, inhibition of neurotransmitter synthesis as well as neurotransmitter reuptake and release [58, 59]. A bi-directional relationship was postulated, which implies that inflammation modifies the susceptibility to unipolar depression [60]. Chronic inflammation has been postulated as a marker to facilitate the detection of unipolar depression as well as suicidal ideation [61]. Persistent inflammatory processes determined by increased CRP and IL-6 levels are also associated with a lack of response to antidepressants [62–65]. Consistent with this, a randomized controlled trial showed that the addition of an NSAID to psychiatric medication improved treatment response [66]. Also, other anti-inflammatory agents like celecoxib and acetylsalicylic acid curcumin and omega-3 fatty acids are investigated as therapeutic agents [67]. An anti-inflammatory factor is Alpha-1-Antitrypsin (AAT-1), which is a glycoprotein that acts as an inhibitor of various enzymes. In macrophages, AAT-1 inhibits the production of tumor necrosis factor-α and matrix metalloproteinase-12, thus acting as an antagonist of pro-inflammatory markers [68]. AAT-1 was found to be significantly higher in patients suffering from unipolar depression [61]. Consistent with these findings, the severity of an AAT-1 deficiency correlates with an increase of anxiety and depressive symptoms [69].

#### Remaining etiological questions

**Fig. 1:**
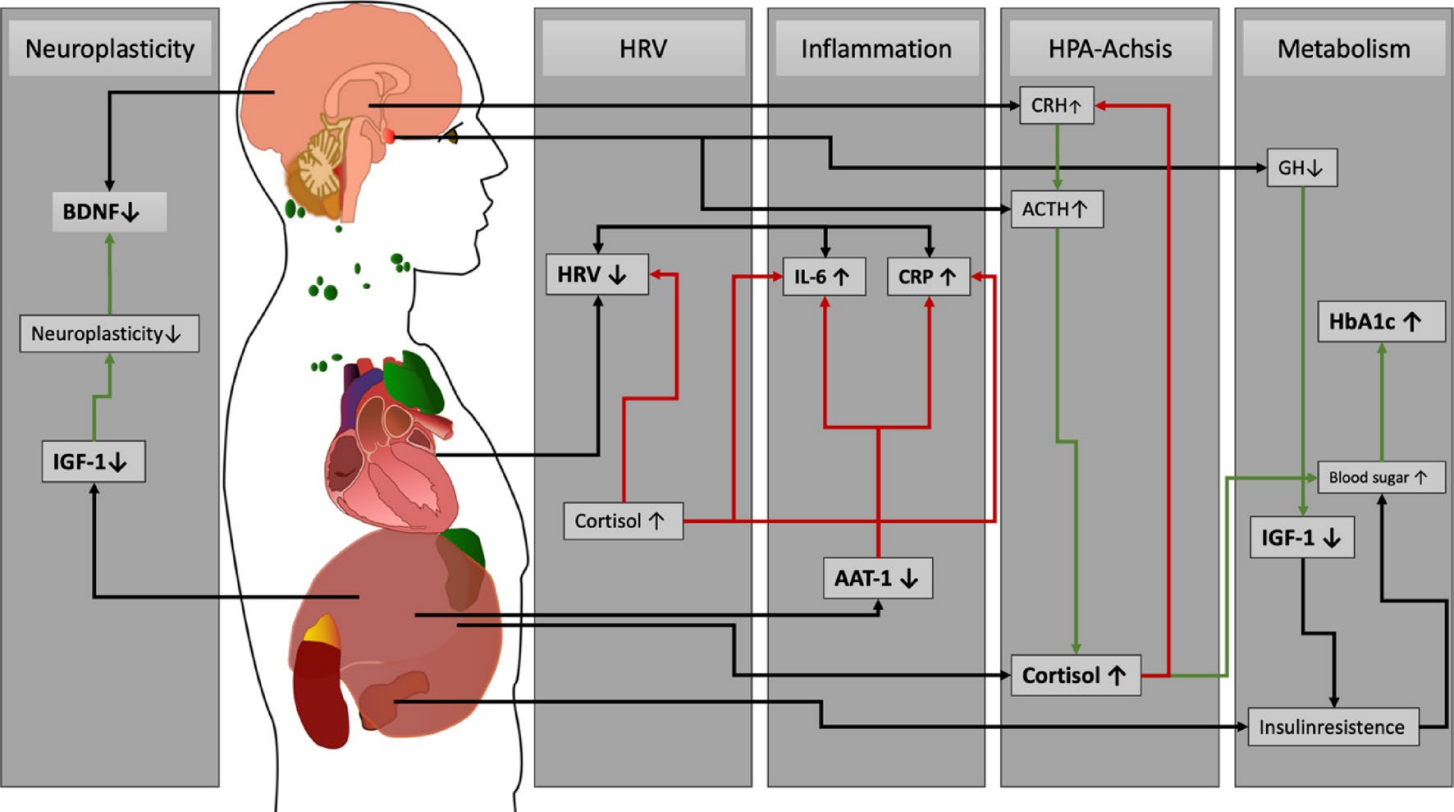
Hypothesized interplay of biological factors in unipolar depression. In this diagram, the interconnectedness of various pathways that are involved in unipolar depression is graphically presented. Negative feedback loops are highlighted with red arrows, while positive feedback is indicated by green arrows. Stress triggers a hormonal cascade, starting with elevated CRH (Corticotropin-Releasing Hormone), which leads to increased ACTH (Adrenocorticotropic Hormone) production, ultimately resulting in the release of Cortisol. High Cortisol levels reduce HRV (Heart Rate Variability) and facilitate the release of inflammatory markers like IL-6 and CRP. In depressed individuals, reduced AAT-1 levels hinder the proper reduction of inflammatory interleukins. Changes in the expression of neurotrophic factors, such as BDNF and IGF-1, have been associated with abnormal neural plasticity. Growth hormone (GH) stimulates the liver to produce IGF-1. IGF-1 also plays a role in insulin homeostasis. Low IGF-1 levels are associated with depression which lead to increased blood sugar levels as well as HbA1c levels

The aforementioned factors, including inflammation, the dysregulation of the ANS and HPA axis, as well as metabolic implications, form an intricate interplay of various factors as illustrated in Fig. 1. Nevertheless, the true interactions among these aspects remain enigmatic, and a comprehensive concept of the biological underpinnings has yet to be unveiled [17, 22]. Through a comprehensive and collective investigation of these factors, our research strives to shed light on these lingering questions, with the goal of contributing to a broader understanding of unipolar depression.

### Study aims and hypotheses

The goal of the POKAL-PSY study is to find relevant risk-factors and predictors determining the severity of unipolar depression in an outpatient setting with a data driven approach. In Addition, easily retrievable parameters should be used to facilitate the diagnostic process in an outpatient setting. To achieve this, we will use clinical parameters as well as a wide array of biological markers such as IGF-1, cortisol, AAT-1, and HRV measurements. We will recruit three cohorts consisting of outpatients with depression at inclusion, patients in primary care without psychiatric disorders, as well as healthy controls with no prior medical history. Moreover, in a cross-sectional analysis, we are aiming to determine whether we can use data-driven algorithms to identify new relevant sub-categories of unipolar depression that are not yet included in current classification systems. In a further step we would investigate the outcomes of each subcategory.

Accordingly, we assume:

1. Functional and structural physiological correlates exist (autonomic nervous system, blood count, inflammatory parameters, fat distribution patterns) that are altered in the presence of depression compared to healthy, or somatically ill but not depressed patients.
2. Physiological and phenotypic differences exist within the overall population of all depressed patients, which influence the outcome and further course of the disease.

## Methods and design

The POKAL-PSY study is a prospective study with three cohorts, where we recruit one cohort with healthy controls (*n* = 50), patients with depression (*n* = 475) and participants without depression (*n* = 425) where participants are monitored for five years. Notably, our emphasis on recruiting individuals with and without depressive symptoms serves to comprehensively investigate the predictors and underlying mechanisms of unipolar depression. The inclusion of patients with somatic comorbidities, who frequently seek medical care, highlights a real-world scenario. For this project, participants will be recruited in a naturalistic setting. Cardiovascular factors (heart rate variability, pulse, blood pressure), metabolic factors (blood lipids, HbA1c, body fat percentage, fasting glucose, IGF-1), as well as inflammatory markers (CRP, AAT-1) will be collected. In addition, the burden of somatic symptoms, lifestyle factors (Eating behavior, chronotype, physical activity), psychopathological characteristics (depression subtype, childhood trauma, anxiety) will be assessed by questionnaires. Depressive relapses will be assessed anamnestically if they occur during the follow-up visits. Patients will be mainly recruited in general practitioner (GP) offices, affiliated with the Bavarian Research Practice Network. Currently, we are recruiting at three different sites. By the end of the recruiting process, our goal is to have recruited from 5 to 6 different sites. We plan to recruit 90% of our study cohort within several GP offices. The inclusion and exclusion criteria are checked in consultation with the treating GPs and in direct patient contact by the study staff and the patients' written consent is obtained. In addition, participants will be recruited at the psychiatric outpatient clinic of the university clinic in Munich. In the context of the POKAL project we cooperate with other research groups and projects by providing validation datasets and support concerning participant recruitment. Specifically, we will cooperate with the MIP3 project (project nr. 21–0357, German Clinical Trials Register number: DRKS00025946). The healthy controls recruited will consist of motivated German citizens. All study personnel were trained to conduct the HRV measurements, as well as the observer ratings. Organizational project management is performed by the Studienzentrum Psychiatrie (SZP) of the LMU Munich. All participants sign a written informed consent form prior to participation. The study was approved by the local ethics committee (project nr. 22–0637, date 14.09.2022) and registered at ClinicalTrials.gov (NCT05547711) and at the German Clinical Trials Register (DRKS00030203). The study presented here is a subproject of the research network POKAL (Predictors and Clinical Outcomes in Depressive Disorders; GRK 2621), which is sponsored by the German Research Foundation (DFG) [70]. Furthermore, all the participants are kindly asked whether they give written consent to participate in the Munich Mental Health Biobank, as well [71]. The POKAL-PSY study is performed in accordance with the regulations of good clinical practice and the declaration of Helsinki [72]. The eligibility criteria can be seen in Table 1.

**Table 1:** Overview of the POKAL-PSY study

**Study goal**
Development of a machine learning algorithm based on relevant risk-factors and predictors to facilitate the diagnosis of depression
**Study design**
Prospective Study with three cohorts
**Inclusion criteria**
Sufficient ability to speak and understand German
Female and male subjects between 18–70 years
Ability to understand and sign the informed consent form
PHQ > 8, if depressed
**Exclusion criteria**
Cognitive limitations that interfere with reliable completion of questionnaires
Presence of a manic episode, bipolar disorder, schizophrenia spectrum disorder, active eating disorder, drug or alcohol dependence syndrome
Presence of uncontrolled systemic disease, uncontrolled somatic (other than metabolic or cardiovascular) /neurologic disease, current or recent (last month) trauma or infectious disease
Patients with acute suicidal ideation
Alpha-1-Antitrypsin deficiency
Pregnant or lactating women
**Discontinuation criteria**
Patients will be excluded if they develop an exclusion criterion during the course of the study. Data collected up to that point will be retained. The data collected up to that point will be retained in anonymized form, unless the participant requests deletion before ceasing their study participation

Table 1: This table provides an overview of the inclusion/exclusion criteria and the key data of the POKAL-PSY study.

### Study timeline

We will perform a follow-up assessment 4 weeks after the participants’ inclusion into the study, and then annually for a total of 5 years, as indicated in Table 2.

**Table 2:** Study assessment overview

	Study inclusion	After 4 weeks	Yearly follow up until 5 years
Observer rating	MADRS, GAF SIMPAQ, expert rating, SCID-CV	MADRS, GAF SIMPAQ	MADRS, GAF SIMPAQ, expert rating,
Self-rating questionnaires	Large questionnaire profile^1^	Small questionnaire profile^2^	Large questionnaire profile^1^
HRV measurements	At least 10 min (5 min supine/sitting each)	At least 10 min (5 min supine/sitting each)	At least 10 min (5 min supine/sitting each)
Bioimpedance scale	Tanita scale	Tanita scale	Tanita Scale
Laboratory assessments	Multiplex analysis, blood collection^3^	Multiplex analysis, blood collection^3^	Blood collection^3^
Vital parameters	Blood pressure, temperature, pulse	Blood pressure, temperature, pulse	Blood pressure, temperature, pulse

Large questionnaire profile^1^: socio-demographic data, family history, medical history, PHQ-9, PHQ-15, MCTQ, GAD-7, postal code, PID5BF + M, WHO-DAS, FEV, CTS, CTQ, PC-PTSD, PACIC, FEV, WHO-5, LSNS 6, BRS, UCLA LS 3, SSD-12, IDS-SR small questionnaire profile^2^: HTQ, PHQ-9, PHQ-15, MCTQ, GAD-7, WHO-DAS, FEV, SSD-12, IDS-SR Blood Collection^*3*^: fasting glucose, triglycerides, morning cortisol, CRP, AAT-1, IGF-1, cortisol, TSH, complete blood count, HbA1c, liver values

### Medical examinations

#### Clinical assessments

Depressive symptomatology is assessed using self-rated instruments (PHQ-9), the Inventory of Depressive Symptomatology (IDS-SR) [73] and observer ratings (MADRS) at inclusion and each follow-up [74]. At inclusion functionality will be rated using the General Assessment of Functioning (GAF) [75, 76]. To precisely detect possible exclusion criteria a SCID-5-CV rating is performed at study inclusion. All assessments are done by experienced and trained raters [77]. Furthermore, a rating is conducted where the raters need to describe their own impression concerning prognosis and give an estimate concerning the disease trajectory.

The abdominal circumference, as well as the hip circumference are measured standing with the upper body free with a measuring tape according to the guidelines of the WHO [78]. Blood pressure measurement (systolic, diastolic) and pulse is performed after 3–5 min of rest while sitting [79]. In addition, body temperature is determined with a tympanic thermometer in the morning [80].

Heart rate and HRV are measured using special chest straps (EcgMove4) from Movisens (Movisens GmbH), which determines body position. A HRV measurement is performed at each follow-up. The participants are kindly asked to be in an upright position for 5 min and lie for 5 min while breathing at a normal pace to achieve a resting state measurement [81]. If needed, participants can take breaks. Each participant should wear the belt at least for 10 min. Healthy controls will wear the belt for 24 h5 if possible. Blood is drawn between 8:00–10:00 in the mornings and analyzed at the Institute of Laboratory Medicine (LMU Hospital). Specifically, glucose, liver values, cholesterol (HDL and LDL), triglycerides, cortisol, TSH, AAT-1, IGF-1, CRP, and complete blood count are measured. The participants are asked not to smoke and to conduct an overnight fast before the examination. If participants are additionally willing to take part in the Munich Mental Health Biobank Project (MMBH), [82] further blood is drawn (9 ml Serum, 9 ml Li-Heparin, 7.5 ml EDTA, 2.4 ml RNA Extract) and stored at – 80 °C. Further analysis of MMHB blood samples is possible in following projects.

The participants are placed on a calibrated body analysis scale of the model TANITA MC-780MA-N (Tanita Corp., Tokyo, Japan) at the time of inclusion and at each subsequent follow-up. This scale uses state-of-the-art multi-frequency segmental bioelectrical impedance analysis (BIA) technology [83]. This technique is especially useful for providing an instant analysis of the participant’s health and fitness status and monitoring their progress over time [84]. Since food ingestion, regardless of macronutrient content, alters the BIA body composition estimates, patients are weighed after an overnight fast [85]. Furthermore, the participants are asked to take off their shoes as well as socks and wear light clothing. The body scale provides measurement parameters such as total body fat in kg and %, trunk fat mass in kg and %, total body muscle mass in kg and % (incl. arm and leg muscle assessment), total body water in kg and % (extra- and intracellular), visceral fat score, body mass index, basal metabolic rate in kcal and kJ, to record the subjects’ fitness and health [86]. To determine the BMI of the study subjects, their height is asked anamnestically.

#### Self-rating questionnaires

Table 2 shows which self-ratings are performed at each follow-up. To increase synergies patients are asked to fill out the Munich Mental Health Biobank Project (MMHBP) questionnaire battery as part of the POKAL-PSY study at inclusion [82].

Eating and sleeping habits will be assessed by the German version of the Three-factor-Eating-Questionnaire (FEV) [87] and the Munich Chronotype Questionnaire (MCTQ) questionnaire [88]. The simple physical activity questionnaire (SIMPAQ) will be taken to examine daily-life physical activities [89]. Also, participants will be asked about their drinking habits and whether they are smoking. Personality and anxiety traits, as well as somatic symptomatology will be measured using the Personality Inventory for DSM-5 Brief Form Plus (PID5BF + M) questionnaire [90], the Somatic Symptom Disorder-B Criteria Scale (SSD-12) [91], the Patient Health Questionnaire 15 (PHQ-15) [92], as well as the 7-item Generalized Anxiety Disorder Questionnaire (GAD-7) [93]. Personal history contains assessment of trauma with the childhood trauma questionnaire (CTQ) [94] and the Primary Care PTSD Screen for DSM-5 (PC-PTSD) [95], and family history, as well as medical history of psychiatric disorders will be gathered. The experienced loneliness is captured by the Lubben social network scale (LSNS 6) [96], as well as the UCLA 3-Item Loneliness Scale (UCLA LS-3) questionnaire [97]. Perceived Resilience and Wellbeing are measured by the WHO- 5 Well-Being Index [98] and the Brief resilience scale (BRS) [99]. Functional Outcomes are rated by performing the general assessment of functioning (GAF) [100] and participants are given the World Health Organization disability assessment schedule 2.0 (WHODAS 2.0) [101].

Table 2: Shows all planned examinations during the study period.

### Data and sample processing

The collected data in the POKAL-PSY study is captured in CentraXX (Kairos GmbH, Bochum, Germany), a laboratory information management system, which has already been used in other research projects at the department of psychiatry in Munich [102, 103]. CentraXX allows the collection and management of all relevant data of study participants [71]. The pseudonymization of the data is guaranteed by the CentraXX application [104].

### Outcome assessment

#### Primary outcome

Our primary outcome is the development of an algorithm that predicts the severity of unipolar depression with biological markers. We will use the routine blood laboratory markers, as well as the HRV data and only include single items concerning the self-rating questionnaire, if necessary. We hypothesize that IGF-1 and AAT-1 will be biological markers that facilitate diagnosing unipolar depression and hence will mainly drive the prediction together with the HRV data. We will use the PHQ-9 and the MADRS at baseline and follow-up to measure changes in depressive symptomatology as well as their severity [48]. Supervised algorithms like decision trees and support vector machines will be used to determine which factors drive the severity of depression at inclusion.

#### Secondary outcomes

Furthermore, we will implement a prospective algorithm predicting the severity of the participants' depressive symptomatology after one year. The results will be compared with the expertrating conducted at baseline. To ensure generalizability we will test the cross-sectional algorithm on the follow-up data. Further we will conduct a structural equation model (SEM) analysis at inclusion to better understand the underlying mechanisms that drive unipolar depression. Unsupervised cluster algorithms will be used to detect biomarker driven subtypes.

#### Mediating variables

Lifestyle factors, personality traits, and personal history are considered to be mediating variables. We will consider blood pressure, medication, age, and gender as confounding factors. The breathing rate is not monitored [105].

### Power calculation

An a priori power analysis was performed to calculate the minimum of needed study participants to conduct a SEM analysis. We assumed a power (1-β) of 0.90 with an effect size of 0.15 and an alpha of 0.05. With these settings a sample size of at least 870 was required to achieve a robust model with 5 latent variables. With estimated screening failures of slightly less than 10% (e.g. development of bipolar disorder in the further course, or with inclusion concealment of a dependence illness) we aim to recruit 950 participants at inclusion. We aim to recruit a sample size of 475 patients with depression and 475 participants without depression. Recruitment in the project presented here will end when the sample size of 950 subjects is reached. Also, this is a sufficient number of participants for machine learning algorithms [106].

### Planned data analyses

Categorical data will be one-hot-encoded if suitable and summarized with percentages and confidence intervals. For ordinal data the median will be given as well as the interquartile range and for continuous data we calculate the mean as well as the standard deviation and the confidence intervals. Demographic and baseline characteristics in the study arms will be compared with suitable statistical tests. Since multiple comparisons will be performed significant *p*.values will be reported false discovery rate corrected [107]. With the cross-sectional data at inclusion a SEM analysis will be conducted [108]. Furthermore, we will investigate the predictive value of baseline biological parameters for change in depressive symptomatology. In addition, we will design a classification algorithm using all relevant data detecting which participant is depressed. This approach involves the implementation of multiple supervised machine learning algorithms to thoroughly assess their performance. We will evaluate each algorithm during the analysis stage and subsequently select the one that yields the most favorable results. These algorithms may include, but are not limited to, support vector machines, decision trees, and neural networks. Each of these algorithms will be carefully applied and evaluated in the context of our research question. Furthermore, we will use multimodal stacked algorithms to assess the generalizability of different clinical questionnaires and biomarkers separately. To ensure the robustness of our algorithm selection and model performance assessment, we will employ a leave-site-out nested cross-validation approach in each model. This mitigates the risk of overfitting and ensures generalizability. Since the eventual goal of our algorithm is the implementation in a clinical context and to guide general practitioners, we plan to develop a sequential prediction model, which reduces the number of required data modalities and diagnostic uncertainty, as described by Koutsouleris et al. [109]. Sequential stacking models can be used to reduce the data required to produce optimum prediction scores. This method can greatly increase clinical applicability since physicians will be only asked for additional information if needed to achieve satisfactory predictive accuracy [109].

Further, we recognize the necessity of employing cluster analysis techniques to gain insights into the heterogeneous nature of depression [17]. It is likely that depression does not encompass a single biological phenotype but rather multiple distinct profiles. To comprehensively address this, we will utilize various clustering algorithms. Our primary clustering algorithm will be non-negative matrix factorization [110]. Subsequently, we will select the clustering algorithm that best aligns with the clinical realities reflected within the identified clusters.

## Study progress

Recruitment for the POKAL-PSY Study began on October 25, 2022. As of September 1, 2023, a total of 230 participants have been enrolled. The recruitment of new patients is anticipated to conclude by October 15, 2027. The current protocol version is 3.0, last revised on July 25, 2023.

**Fig. 2:**
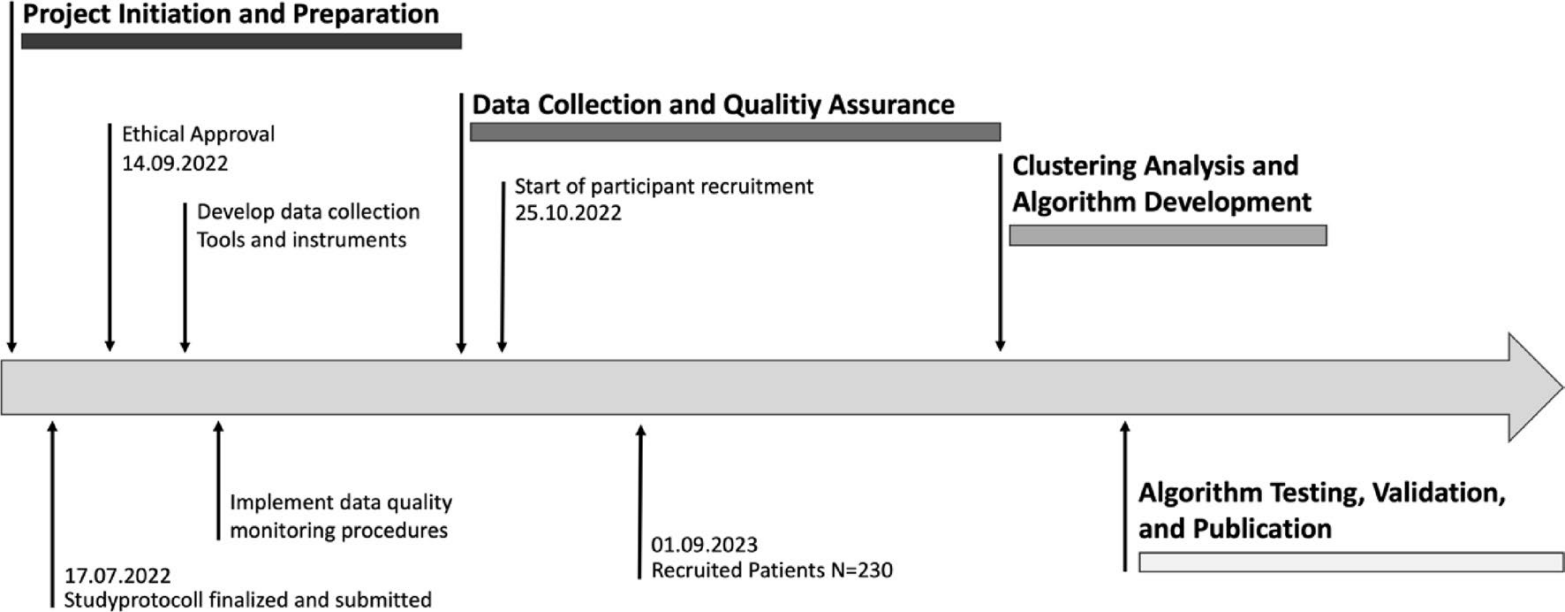
POKAL-PSY study timeline

Figure 2: This figure outlines the strategic plan for the POKAL-PSY project, detailing key phases and milestones

## Dissemination

The results of the trial will be analyzed and published after analysis. The results will be reported in the first instance to the study collaborators and the DFG. A summary of the study findings will be made available on the POKAL website (www.pokal-kolleg.de). Scientific publication of the results will be attempted in peer reviewed journals. Further, a dissemination concerning research results will be enabled by the POKAL network and associated programs.

## Discussion

The search for biomarkers to diagnose depression is an endeavor being pursued in psychiatric research since the 1980s [111], but barely resulted in application in clinical practice. The successful application remains challenging since the symptomatology of depression is very heterogeneous and on the other hand because there is no diagnostic gold standard that focuses on an underlying biological mechanism [112]. Despite knowing about the metabolic, endocrine, inflammatory as well as autonomous dysregulation that has been observed in depressed patients, none of these are broadly used to stratify patients. For example, cortisol is a well-studied blood biomarker that has been found to predict an onset or relapse of unipolar depression [113]. Nevertheless, measuring cortisol barely plays a role in clinical practice. One possible reason is that changes in the HPA axis are not specifically found in unipolar depression [114]. Further, many studies found contradicting results which might be due to the heterogeneous nature of the disorder [38]. Patients with the diagnosis of “unipolar depression” are phenotypically, genetically and biologically differing, eventually impacting any biological approaches to better understand depression [115]. Furthermore, exploring potential biomarkers mostly cross-sectional studies were performed which are inherently unable to distinguish consequences secondarily to the illness from alterations caused by the disease itself [116]. Prospective evidence is dearly needed to explain onset and recurrence of unipolar depression. Through machine learning approaches as well as SEM analysis interactions between biomarkers can be examined instead of investigating biomarkers separately [117].

### Translational psychiatry and precision psychiatry

Numerous approaches to improve psychiatric diagnostics and therapy can already be found in the scientific literature. Nevertheless, there is only slow progress concerning translation of psychiatric findings into practice, which is in literature often referred to as the “translational gap” [118]. Health care professionals often do not know what positive impact the implementation of new programs will have on their patients, with datasets that are mainly gathered in hospitals and rarely in an outpatient setting [119, 120]. Often MRI imaging and expensive biomarkers are supposedly needed to implement current findings which are usually too cost-intensive to retrieve in primary care [121]. This led us to focus on biomarkers which are easy to retrieve in an outpatient setting. Laboratory testing of Cortisol, TSH, AAT-1, IGF-1 and CRP is usually possible in an outpatient setting. IGF-1 is measured for the evaluation of hormone status and is thus also frequently part of the standard services of laboratory medical facilities. Making the panel a scalable blood-biomarker panel to investigate in an outpatient setting. Similarly, HRV measurements can be easily obtained in an outpatient setting. Commercially available long-term ECG devices can typically also measure HRV, and report stress-associated parameters derived from it. Hence, this set-up reduces translational barriers concerning later implementation [122]. Since several biologically detectable disease patterns presumably can be captured under the guise of depression, it is important to also consider the interactions of relevant factors. SEM models as well as several machine learning algorithms are suitable for this purpose, since they allow researchers testing direct as well as indirect relations and take several variables into account, making it a statistical model which could provide an alternative perspective at biomarkers and their interaction in psychiatric research [123]. With machine learning approaches also non-linear associations can be explored without the need to pre-define hypotheses, enabling a broader look into different topics.

### Development of an algorithm to detect unipolar depression

The study presented here can be used to establish an algorithm that is supposed to facilitate the diagnosis of unipolar depression in an outpatient setting. Such a tool would be particularly valuable because most studies of unipolar depression are conducted primarily in the hospital setting. Currently, only about half of depressed patients in an outpatient setting are detected, where again only a fraction receives treatment [10]. Current screening rates for unipolar depression in primary care are low, despite the high socioeconomic burden, emphasizing the dire need of fast and easy to use algorithms [124]. In a further step, validation of the model by a follow-up study would be necessary [125]. Prognosis of affected people worsens if their symptomatology goes undetected for a long time [126]. Regarding the high lifetime prevalence as well as the probability of a relapse, routine screening data indicate that a routine screening for unipolar depression is cost-effective [127]. Several barriers were identified in a qualitative study. First, primary care physicians could not adequately perform the screenings due to a lack of time, and second, there was a lack of capacity to provide patients with the ideal treatment. Third, it was also observed that, due to stigmatization, patients were only honest about their symptoms if they had established the necessary basis of trust with their family doctor [124]. Having said this, the algorithm could help primary care physicians to overcome those boundaries more easily. In 2021 Fusar-Poli et al. described the potential of preventive psychiatry to profoundly improve mental health services in our society, since currently mental health services are not focussing on the prevention of unipolar depression or other mental disorders. A “one-size fits all” approach is, according to Fusar-Poli, not effective; he stresses the need for precision psychiatry together with preventive measures in a primary care setting. Often patients at risk show rather nonspecific symptoms impeding early detection in a primary care setting [128]. Through effective stratification, precision psychiatry will significantly improve medical care for individuals [129].

### Strengths and limitations of this study

The presented study has particular value since especially in Germany, outpatient samples are rare. Even more so if they are longitudinal and prospective. As a result, the comprehensive outcome measures will provide unique information about physical and emotional health of the participants. Commonalities and differences in transition pathways and outcomes will contribute to identifying groups of patients who might benefit from differing treatment approaches or who might need more intensive care early on to improve the long-term outcome. Since a prospective period of 5 years is long and the medical examinations are only annually there might be high dropout rates, because participants might move to other places or might feel less willing to participate in our study, potentially leading to an attrition bias [130]. Since healthy participants will also be recruited by flyers, especially trained and weight conscious participants might be attracted to the study because of the usage of the bioimpedance scale, potentially leading to a sampling bias.

## Data Availability

We aim to make anonymized data available to other research teams interested in further exploration and analysis upon request.
